# Simultaneous implant placement with autogenous onlay bone grafts: a systematic review and meta-analysis

**DOI:** 10.1186/s40729-021-00311-4

**Published:** 2021-04-30

**Authors:** Guoqiang Ma, Chaoan Wu, Miaoting Shao

**Affiliations:** 1 Department of Oral Implantology, Jinhua Stomatological Hospital, 277 Silian Rd, Jinhua, 321000 Zhejiang Province People’s Republic of China; 2 Department of Stomatology, Yingtan Shangpin Dental Clinic, Yingtan, 335000 Jiangxi Province People’s Republic of China

**Keywords:** Block grafts, Iliac graft, Survival, Immediate placement, Ridge augmentation

## Abstract

Several authors have suggested that implants can be placed simultaneously with onlay bone grafts without affecting outcomes. Therefore, the purpose of this study was to answer the following clinical questions: (1) What are the outcomes of implants placed simultaneously with autogenous onlay bone grafts? And (2) is there a difference in outcomes between simultaneous vs delayed placement of implants with autogenous onlay bone grafts? Databases of PubMed, Embase, and Google Scholar were searched up to 15 November 2020. Data on implant survival was extracted from all the included studies (single arm and comparative) to calculate point estimates with 95% confidence intervals (CI) and pooled using the DerSimonian–Laird meta-analysis model. We also compared implant survival rates between the simultaneous and delayed placement of implants with data from comparative studies. Nineteen studies were included. Five of them compared simultaneous and delayed placement of implants. Dividing the studies based on follow-up duration, the pooled survival of implant placed simultaneously with onlay grafts after <2.5 years of follow-up was 93.1% (95% CI 82.6 to 97.4%) and after 2.5–5 years was 86% (95% CI 78.6 to 91.1%). Implant survival was found to be 85.8% (95% CI 79.6 to 90.3%) with iliac crest grafts and 95.7% (95% CI 83.9 to 93.0%) with intra-oral grafts. Our results indicated no statistically significant difference in implant survival between simultaneous and delayed placement (OR 0.43, 95% 0.07, 2.49, *I*^2^=59.04%). Data on implant success and bone loss were limited. Data indicates that implants placed simultaneously with autogenous onlay grafts have a survival rate of 93.1% and 86% after a follow-up of <2.5 years and 2.5–5years respectively. A limited number of studies indicate no significant difference in implant survival between the simultaneous and delayed placement of implants with onlay bone grafts. There is a need for randomized controlled trials comparing simultaneous and delayed implant placement to provide robust evidence.

**Supplementary Information:**

The online version contains supplementary material available at 10.1186/s40729-021-00311-4.

## Background

Several authors have suggested that implants can be placed simultaneously with onlay bone grafts without affecting outcomes. Therefore, the purpose of this study was to answer the following clinical questions: (1) What are the outcomes of implants placed simultaneously with autogenous onlay bone grafts? And (2) is there a difference in outcomes between simultaneous vs delayed placement of implants with autogenous onlay bone grafts?

## Introduction

Rehabilitation of partially as well as completely edentulous patients with dental implants is considered to be the most optimal method to restore esthetics and function with predictable long-term results [1]. However, alveolar ridge defects or completely atrophic alveolar ridges pose a considerable problem for implant rehabilitation in a large number of patients [2]. Bone augmentation procedures such as inlay or onlay grafts, guided bone regeneration, and distraction osteogenesis are usually advised to manage alveolar defects before implant placement to obtain the minimum bony requirement for successful positioning of dental implants [3, 4]. Studies indicate that regeneration procedures offer predictable results, and the success of implants placed in regenerated areas is very similar to that of implants placed in pristine bone [5].

Autogenous onlay bone grafts from intra-oral, iliac, and cranium have been successfully used for ridge augmentation in a number of studies to date [6, 7]. The standard procedure consists of initial bone grafting and a second stage surgery after graft maturation for placement of implants. A waiting period of 3–6 months is usually indicated depending upon the size of the graft, recipient site, and the type of onlay graft [6]. A disadvantage of this protocol is that second stage implant placement delays the prosthetic phase and increases the time of rehabilitation for the patient. In this context, several authors have suggested that implants can be placed simultaneously with onlay bone grafts without affecting outcomes [8, 9]. Researchers have indicated that implant survival may be more dependent on the native bone supporting the implant rather than the grafted bone [9, 10]. In the past, a few systematic reviews have attempted to assess outcomes of simultaneous vs delayed placement of implants with onlay bone grafts [11, 12]. However, these reviews could include very few studies (5–7 in number) and were unable to conduct a meta-analysis of implant outcomes to present clear evidence to practicing clinicians. Therefore, the purpose of this study was to answer the following clinical questions: (1) What are the outcomes of implants placed simultaneously with autogenous onlay bone grafts? And (2) is there a difference in outcomes between simultaneous vs delayed placement of implants with autogenous onlay bone grafts?

## Material and methods

### Inclusion criteria

This systematic review and meta-analysis were conducted as per the PRISMA statement (Preferred Reporting Items for Systematic Reviews and Meta-analyses) [13]. Studies fulfilling the following inclusion criteria were identified: (1) For the first clinical question, we identified all prospective and retrospective studies reporting outcomes of implants placed simultaneously with autogenous onlay bone grafts. (2) For the second clinical question, we identified all prospective and retrospective studies comparing outcomes of simultaneous implant placement vs delayed implant placement with autogenous onlay bone grafts. (3) The included studies were to report data of at least 10 implants. No restriction was placed on the area of the harvest of onlay graft, type of defect, and location of the implant. The following studies were excluded: (1) Studies reporting the use of inlay or sandwich bone graft, (2) studies reporting the use of guided bone regeneration, (3) studies using vascularized bone grafts, (4) studies not reporting implant success or survival, (5) animal studies, review articles, non-English language publications, and case reports. In the case of studies reporting duplicate data, we included the study with the largest sample size.

### Search strategy

Databases of PubMed, Embase, and Google Scholar were searched to identify relevant publications. All databases were screened from inception to 15 November 2020. The search was conducted by two reviewers independent of each other. Keywords used were as follows: “implants”, “dental implants”, “onlay grafts”, “iliac graft”, “block graft” “ridge augmentation”, “calvarial graft”, “intraoral graft”, “autogenous graft”, “simultaneous”, “immediate”, and “one-stage”. The search strategy used in the PubMed database is demonstrated in Supplementary Table S1. Articles in the search results were evaluated by each reviewer by their titles and abstracts. Articles applicable to the review were identified, and their full texts were sourced. Both the reviewers assessed individual articles based on the inclusion and exclusion criteria. Any disagreements were resolved by discussion. Post-screening, the bibliography of included studies was hand searched for any additional references.

### Data extraction and risk of bias assessment

Two reviewers independently extracted data from the included studies. Data regarding authors, publication year, study location, number of patients, number of implants, age, number of smokers, type of onlay graft, defect type, implant location, success criteria, survival and success data, marginal bone loss, recipient site complications, and follow-up were extracted. The primary outcome of interest was the survival and success of implants placed simultaneously with autogenous onlay grafts. The secondary outcome of interest was to compare the implant success and survival between the simultaneous and delayed placement of implants by pooling data from comparative studies.

We assessed the quality of included studies using the method recommended by Clementini et al. [14]. Each study was assessed for random sample selection, the definition of inclusion and exclusion criteria, reporting and monitoring the implant loss, validated measurements, and statistical analysis. Studies reporting on all of these domains were classified as having a low risk of bias, studies omitting any one of the criteria were classified as having a moderate risk of bias, and the remaining studies were assigned a high risk of bias.

### Statistical analysis

The software “Open MetaAnalyst” was used for the meta-analysis [15]. Considering the heterogeneity in the included studies, a random-effects model was preferred for the meta-analysis. The *I*^2^ statistic was used to assess inter-study heterogeneity. *I*^2^ values of 25–50% represented low, values of 50–75% medium, and more than 75% represented substantial heterogeneity. For the primary outcome, data on implant survival was extracted from all the included studies (single arm and comparative) to calculate point estimates with 95% confidence intervals (CI). Data was then transformed using the logit transformation for pooling the proportions using the DerSimonian–Laird meta-analysis model. A sensitivity analysis was conducted to assess the contribution of each study to the pooled prevalence by excluding individual studies one at a time and recalculating the pooled estimate for the remaining studies. Sub-group analysis was carried out based on the duration of follow-up, type of onlay graft used, and type of defect. After a general assessment of follow-up durations of all studies, we grouped studies into follow-up groups of 2.5–5 years and < 2.5 years. Few studies had a slight overlap in the follow-up duration between the groups. Following consultation between the reviewers, it was decided to include studies in the group with which there was a maximum overlap of the follow-up duration. In the second part of the analysis, we compared implant survival rates between the simultaneous and delayed placement of implants with data from comparative studies. The odds ratio (OR) was calculated with 95% CI.

## Results

### Details of included studies

**Fig. 1:**
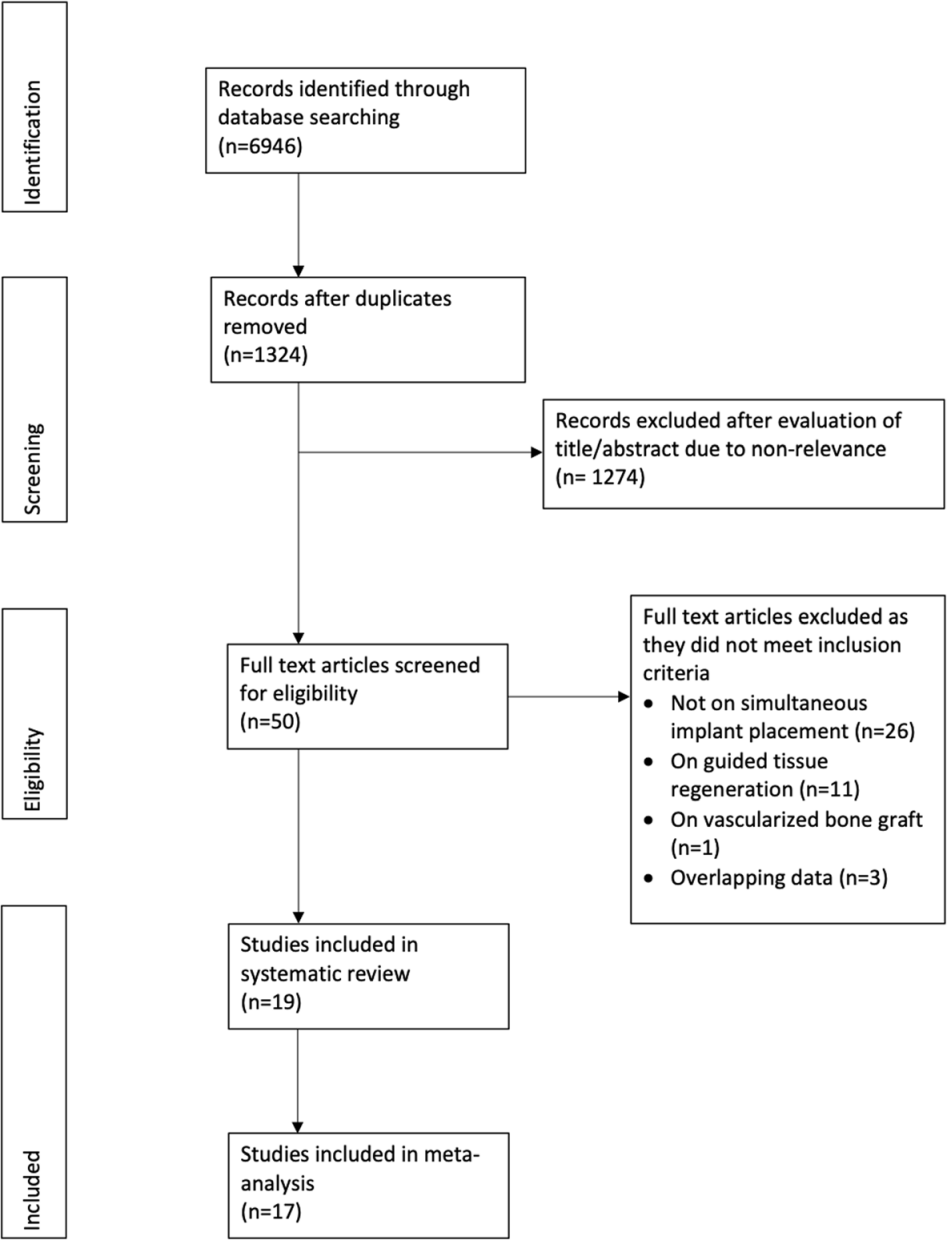
Study flow chart

The study flow-chart is presented in Fig. 1. A total of 19 studies were included in the review with data of 1504 implants [8, 9, 16–32]. Fourteen were single-arm studies assessing only outcomes of implants placed simultaneously with onlay bone grafts [8, 20–32]. Five studies compared outcomes of simultaneous vs delayed placement of implants [9, 16–19].

Details of all single-arm studies are presented in Table 1. Except for three studies [8, 20, 25], all single-arm studies were conducted in European countries. Two were prospective studies [25, 30] while the remaining were retrospective studies. Almost all studies reported the use of iliac and/or intra-oral bone grafts. One recent study used calvarial onlay grafts [20]. Data on the number of smokers was not available in the majority of studies. The number of implants assessed in the studies ranged from 16 to 248, and follow-up ranged from 6 months to 10 years. In all studies, multiple implants were placed in the grafted sites.

**Table 1:** Characteristics of single arm studies

Study	Study location	Study type	Onlay graft donor site	Defect type	Age (years)	Number of patients	Number of implant (patients)	Implant location	Post-loading follow-up	Implant success (%)	Implant survival (%)	Bone Loss from grafted level
Adell et al. 1990 [24]	Sweden	RS	Iliac	NR	NR	23	124	Mx	Mean 4.2 years	NR	73.8	Mean 1.49mm after 1 year
Isaksson and Alberius 1992 [23]	Sweden	RS	Iliac	NR	44–70	8	46	Mx	2.6–5 years	NR	83%	NR
Åstrand et al. 1996 [22]	Sweden	RS	Iliac	V and H	41–70	17	92	Mx	3 years	NR	75	Average 2.6mm
McGrath et al. 1996 [21]	Netherlands	RS	Iliac	V	NR	18	36	Mn	1–2.6 years	91.6	100	9-10%
Vermeeren et al. 1996 [32]	Netherlands	RS	Iliac	V	NR	31	78	Mn	5 years	NR	89.7	50%
Steenberghe et al. 1997 [31]	Belgium	RS	Iliac	V and H	35–68	13	72	Mx	1–10 years	85	NR	Average 1.4mm
Verhoeven et al. 1997 [30]	Netherlands	PS	Iliac	V	49–78	13	30	Mn	Mean 2.4 years	NR	100	36%
Lekholm et al. 1999 [29]	Scandinavia	RS	Iliac and intra-oral	V and H	NR	31	181	Mx	3 years	NR	76	NR
Nyström 2004 [28]	Sweden	RS	Iliac	V and H	39.4–66.8	30	177	Mx	5–13 years	NR	5 year: 74.6 10 year: 72.8	4.8± 0.13mm
van der Meij et al. 2005 [27]	Netherlands	RS	Iliac	NR	37–69	17	34	Mn	Mean 4.3 years	88.2	91	15%
Boronat et al. 2010 [26]	Spain	RS	Intra-oral	H	NR	37	73	Mx and Mn	1 year	95.9^a^	95.9	0.64± NR mm
Kang et al. 2015 [8]	Korea	RS	Iliac and intra-oral	V and H	40–72	33	248	Mx and Mn	3 years	NR	98.38	36.4%
El Zahwy et al. 2019 [25]	Egypt	PS	Chin	V	24–47	8	16	Mx	6 months	NR	81.25	4.77± 1.67 mm
Kablan 2020 [20]	Israel	RS	Cranium	V	20–63	11	63	Mn	2–4.8 years	100	100	NR

*PS* prospective study, *RS* Retrospective study, *V* Vertical, *H* Horizontal, *NR* Not reported, *Mx* Maxilla, *Mn* Mandible

*Albrektsson’s criteria

Characteristics of the five studies comparing outcomes of simultaneous vs delayed placement of implants are presented in Table 2. All were retrospective cohort studies conducted in the USA, Sweden, Spain, and Turkey. Three studies used only iliac grafts [16, 18, 19]; one used iliac and cranial grafts [17] while the remaining study reported the use of intra-oral grafts [9]. The number of implants in the simultaneous placement group varied from 21 to 68 while in the delayed placement group varied from 61 to 147. Follow-up was at least 1 year in all studies. None of the included studies (single arm or comparative) clearly mentioned the criteria or minimum alveolar bone dimensions for simultaneous placement of implants. Peñarrocha-Diago et al. [9] and El Zahwy et al. [25] reported that all patients were preoperatively assessed for the presence of adequate alveolar width to provide primary stability for simultaneous placement of implants.

**Table 2:** Characteristics of comparative studies

Study	Study location	Study type	Onlay graft donor site	Defect type	Age (years)	Smokers (%)	Number of implant (patients)	Implant location	Implant success	Bone loss from grafted level	Timing of implant in DP group (months)	Post-loading follow-up
Misch and Dietsh 1994 [16]	USA	RS	Iliac	V and H	NR	NR	SP 21 (-) DP 147 (-)	Mx Mx	NR	NR	NR	26–97 months
Triplett and Schow 1996 [17]	USA	RS	Iliac and cranium	V and H	NR	NR	SP 65 (NR) DP 110 (NR)	Mx and Mn Mx and Mn	84.6 88.2	NR	4–8	Minimum 1 year
Widmark et al. 2001 [18]	Sweden	RS	Iliac	V and H	NR	NR	SP 68 (NR) DP 33 (NR)	Mx and Mn Mx and Mn	NR	NR	3–4	5 years
Peñarrocha-Diago et al. 2013 [9]	Spain	RS	Intraoral	H	Range 21–82	42.8	SP 38 (20) DP 33 (22)	NR NR	89.5^a^ 96.9	0.69± 0.67 0.20± 0.50	5–8	1 year
Tosun et al. 2017 [19]	Turkey	RS	Iliac	NR	49.3± 11.8	NR	SP 42 (-) DP 61 (-)	13 Mx, 29 Mn 48 Mx, 13 Mn	NR	1.31± 0.95 0.49± 076	3	29 ± 4.2

*NR* Not reported, *V* Vertical, *H* Horizontal, *Mx* Maxilla, *Mn* Mandible, *SP* Simultaneous placement, *DP* Delayed placement

^a^Buser’s criteria

### Outcomes

**Fig. 2:**
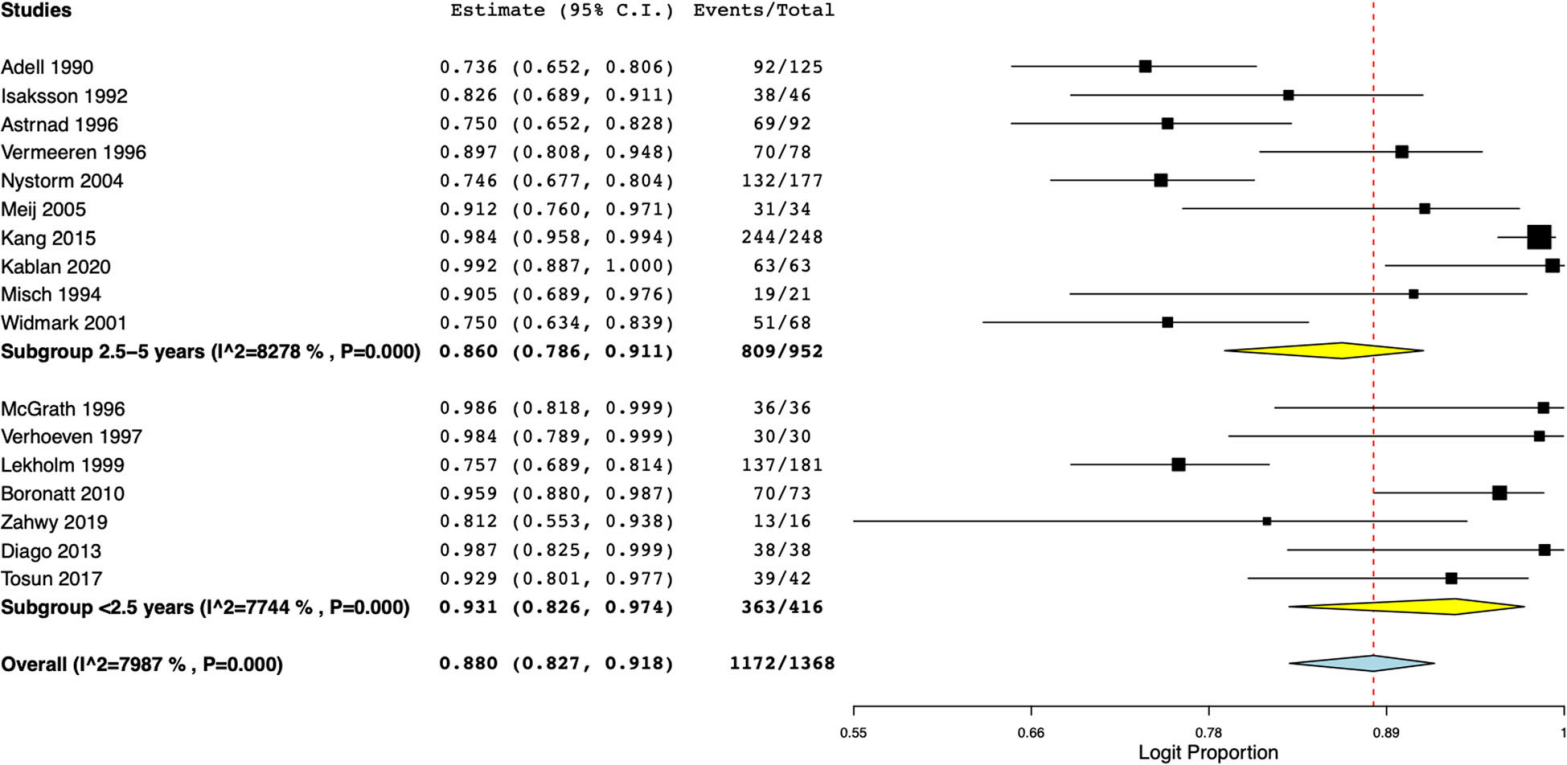
Meta-analysis of implant survival with sub-group analysis based on follow-up duration

**Fig. 3:**
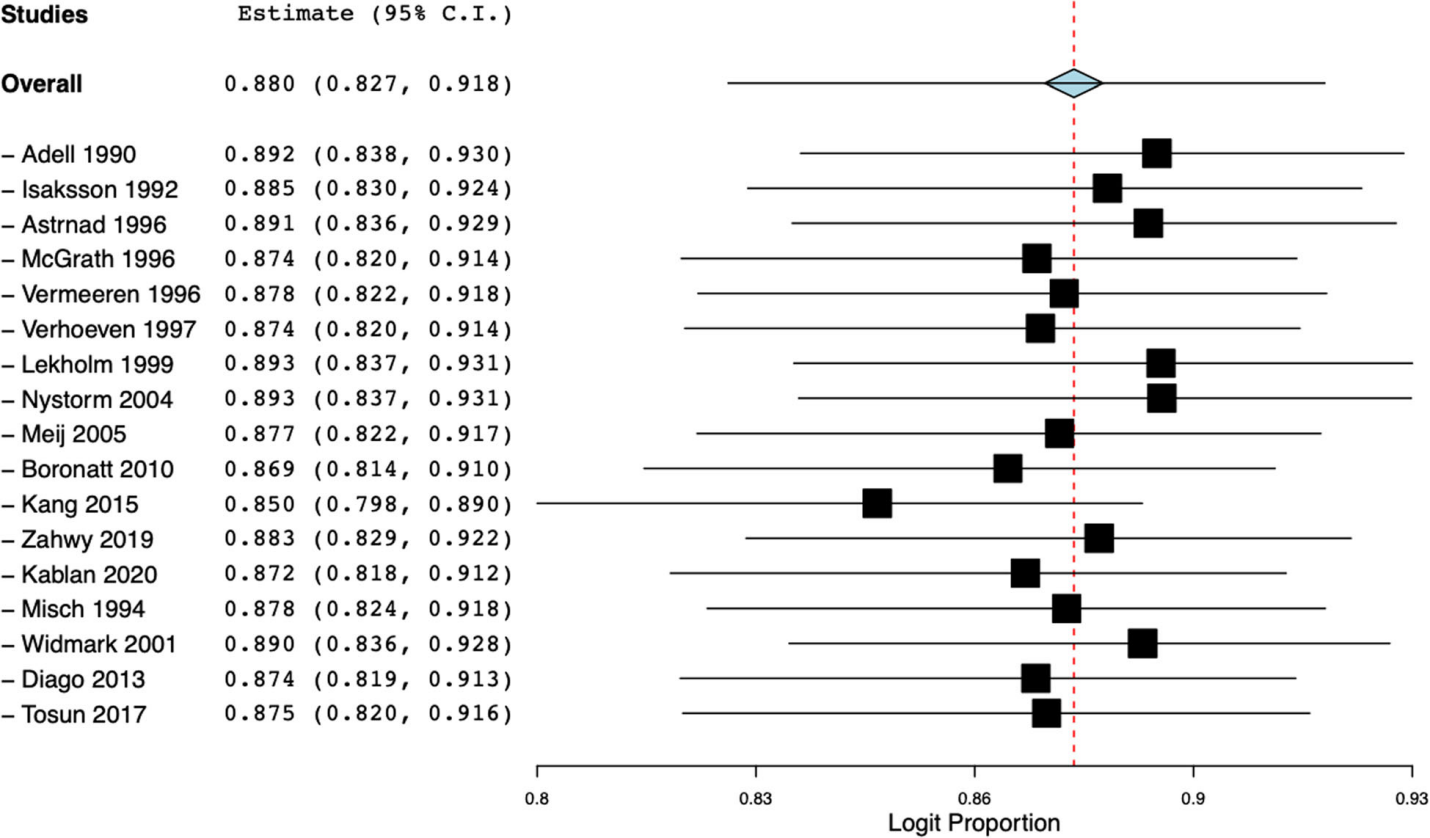
Results of sensitivity analysis presenting pooled implant survival after sequential exclusion of one study at a time

Data on implant survival following simultaneous placement with onlay grafts were available from 17 of the 19 studies. Pooling data of 1368 implants, our meta-analysis indicated an overall survival of 88% (95% CI 82.7 to 91.8%) with a variable follow-up duration of 6 months to 5 years (Fig. 2). Dividing the studies based on follow-up duration, the pooled survival after <2.5 years of follow-up was 93.1% (95% CI 82.6 to 97.4%) and after 2.5–5 years was 86% (95% CI 78.6 to 91.1%). Results of sensitivity analysis are presented in Fig. 3. On sequential exclusion of individual studies, the implant survival ranged from 85 to 89.3%. Only one study reported implant survival after 10 years of follow-up. Nyström et al. [28] in an analysis of 177 implants placed with simultaneous iliac grafts reported implant survival of 72.8%.

**Fig. 4:**
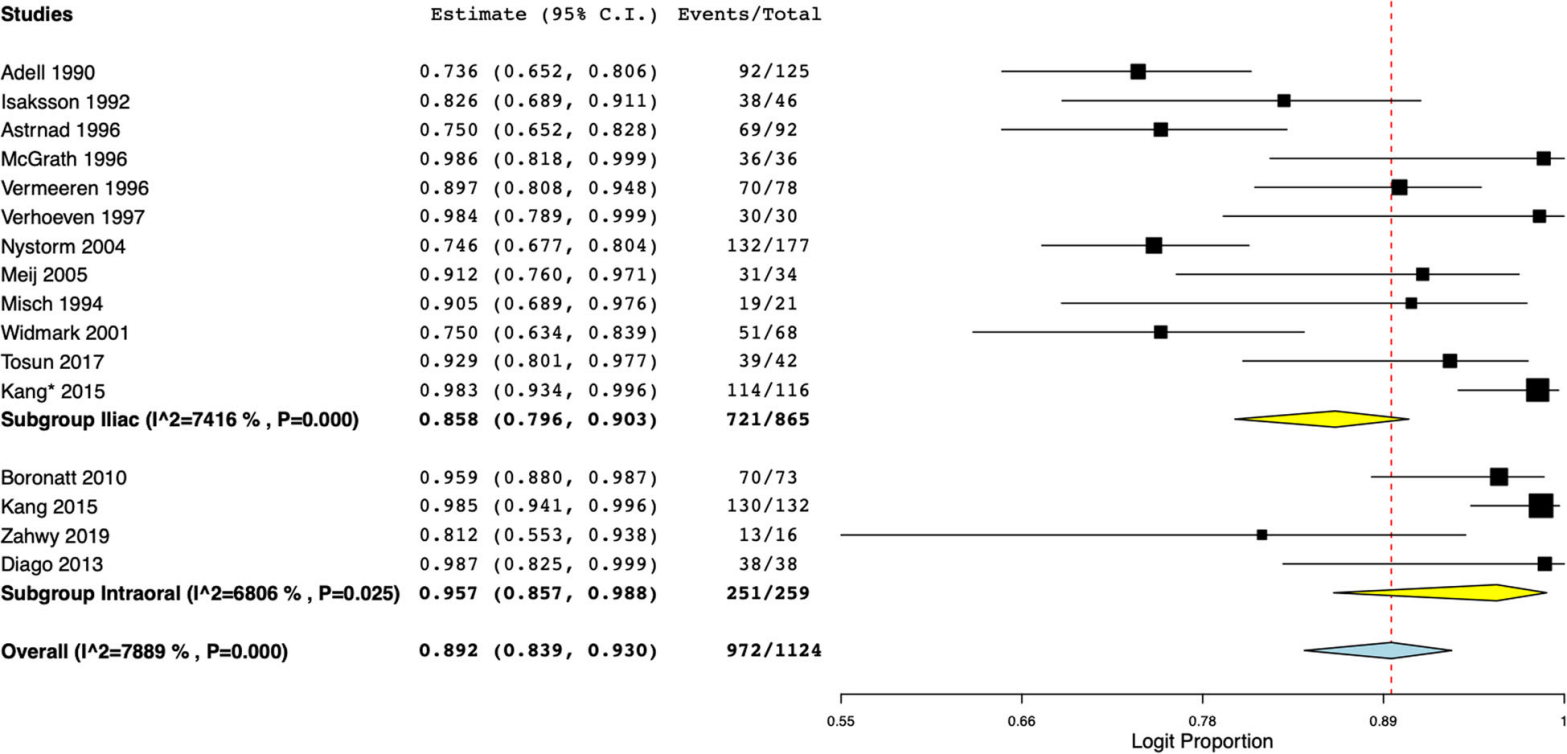
Meta-analysis of implant survival with sub-group analysis based on type of onlay graft

**Fig. 5:**
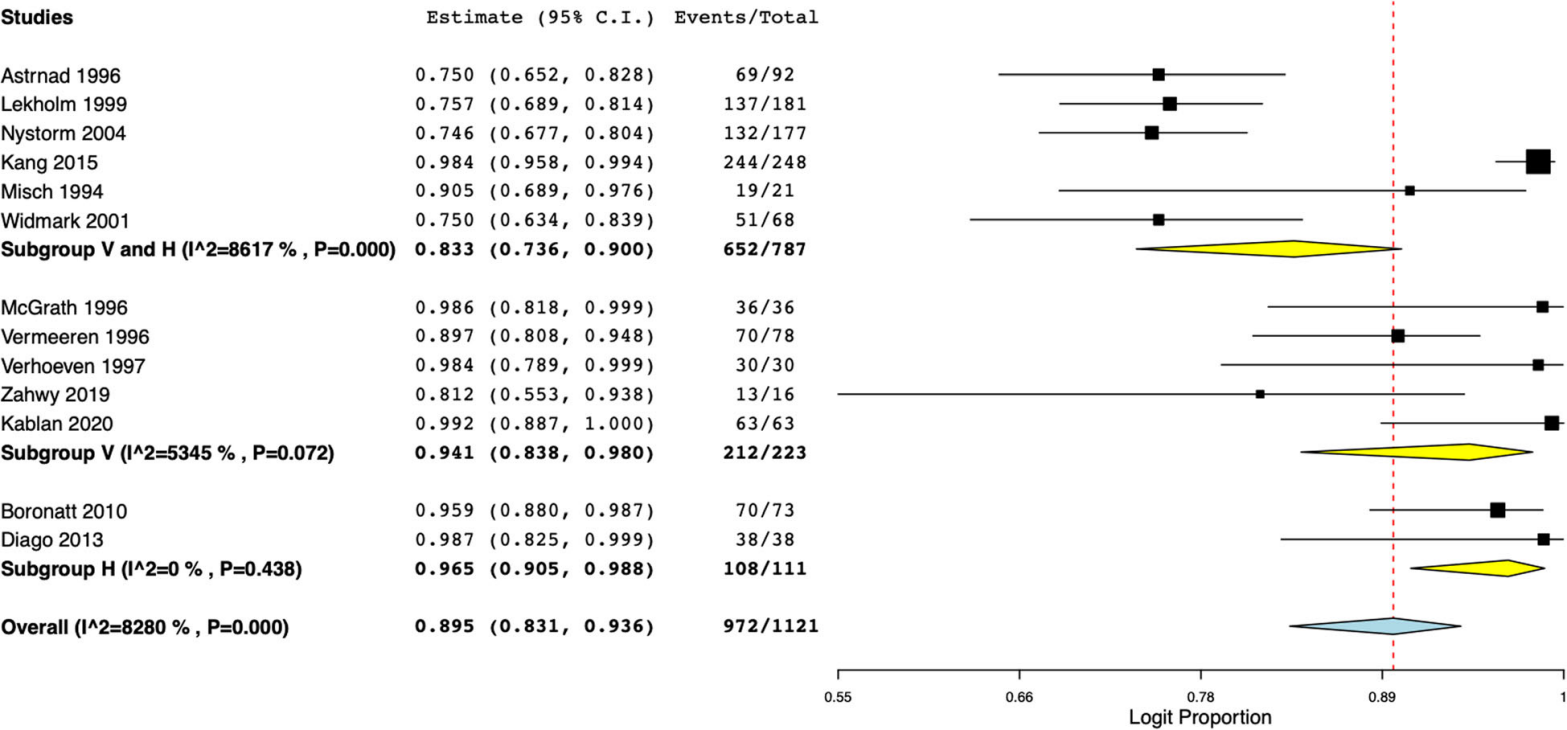
Meta-analysis of implant survival with sub-group analysis based on type of defect. V, vertical defect; H, horizontal defect

We further attempted to analyze implant survival based on the type of onlay bone grafts. Implant survival was found to be 85.8% (95% CI 79.6 to 90.3%) with iliac crest grafts and 95.7% (95% CI 83.9 to 93.0%) with intra-oral grafts (Fig. 4). Based on the type of defect, our analysis indicated implant survival of 94.1% (95% CI 83.8 to 98%) with vertical defects, 96.5% (95% CI 90.5 to 98.8%) with horizontal defects, and 83.3% (95% CI 73.6 to 90%) for both vertical and horizontal defects (Fig. 5). Only seven of the 19 studies reported data on implant success, and only two reported the criteria for success. Therefore, to avoid a biased presentation of results from a small number of studies, we did not perform a meta-analysis for implant success, and data for the same are presented descriptively in Tables 1 and 2.

**Fig. 6:**
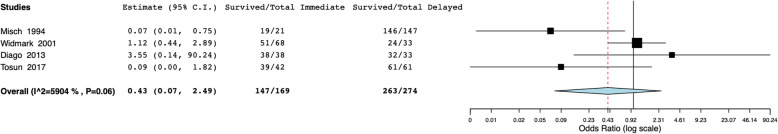
Comparison of implant survival between simultaneously placement and delayed placement of implants with onlay bone grafts

Data on bone loss following grafting was not reported as the mean and standard deviation in all studies. Few studies reported only average values while others reported a percentage of bone loss from grafted levels (Table 1). In the five comparative studies, data on bone loss was available from only two studies, and both reported a significantly higher bone loss in the simultaneous implant placement group as compared to the delayed implant placement group [9, 19]. On comparing implant survival between simultaneous and delayed implant placement with data from four studies, our results indicated no statistically significant difference between the two groups (OR 0.43, 95% 0.07, 2.49, *I*^2^=59.04%) (Fig. 6). Implant success was reported only by two studies, and both indicated no statistically significant difference between the two groups.

Complications following simultaneous onlay grafting and implant placement were reported only by six of the 19 studies. Details are presented in Table 3. Wound dehiscence and graft exposure and graft loss were the most frequent complications with an incidence of 11.7 to 62.5% and 5.4 to 25% respectively. The quality assessment of the included studies is presented in Table 4. Only one study was judged to have a low risk of bias while all others had moderate to high risk of bias.

**Table 3:** Complications related to graft placement in the included studies

Study	Simultaneous
Adell et al. 1990 [24]	Wound dehiscence with graft exposure (5 patients, 21.7%)
van der Meij et al. 2005 [27]	Wound dehiscence with graft exposure (2 patients, 11.7%) Graft loss (2 patients, 11.7%)
Boronat et al. 2010 [26]	Wound dehiscence with graft exposure (8 patients, 21.6%) Graft loss (2 Pts, 5.4%)
Peñarrocha-Diago et al. 2013 [9]	Simultaneous placement group: Wound dehiscence with graft exposure (3 patients, 15%) Wound dehiscence w/o graft exposure (1 patient, 5%) Graft loss (2 patients, 20%) Delayed placement group: Wound dehiscence with graft exposure (4 patients, 18.2%) Graft loss (1 patient, 4.5%) New graft required at implant placement (2 patients, 9%)
Tosun et al. 2017 [19]	Simultaneous placement group: Graft exposure causing failure of three implants Delayed placement group: None reported
El Zahwy et al. 2019 [25]	Wound dehiscence with graft exposure (5 patients, 62.5%) Graft loss (2 Pts, 25%)

**Table 4:** Quality assessment of included studies

Study	Random selection in population	Defined inclusion/exclusion criteria	Reported loss to follow-up	Validated measurements	Statistical analysis	Estimated potential risk of bias
Adell et al. 1990 [24]	No	No	Yes	Yes	Yes	High
Isaksson and Alberius 1992 [23]	No	No	No	No	No	High
Åstrand et al. 1996 [22]	No	No	No	Yes	Yes	High
McGrath et al. 1996 [21]	No	No	No	Yes	Yes	High
Vermeeren et al. 1996 [32]	No	No	No	Yes	Yes	High
Steenberghe et al. 1997 [31]	No	No	No	Yes	Yes	High
Verhoeven et al. 1997 [30]	No	No	No	No	No	High
Lekholm et al. 1999 [29]	No	Yes	Yes	No	No	High
Nyström 2004 [28]	No	No	No	Yes	Yes	High
van der Meij et al. 2005 [27]	No	Yes	Yes	Yes	Yes	Moderate
Boronat et al. 2010 [26]	No	Yes	Yes	Yes	Yes	Moderate
Kang et al. 2015 [8]	No	Yes	Yes	Yes	Yes	Moderate
El Zahwy et al. 2019 [25]	Yes	Yes	Yes	Yes	Yes	Low
Kablan 2020 [20]	No	No	No	No	No	High
Misch and Dietsh 1994 [16]	No	No	No	No	No	High
Triplett and Schow 1996 [17]	No	No	No	No	No	High
Widmark et al. 2001 [18]	No	Yes	Yes	Yes	Yes	Moderate
Peñarrocha-Diago et al. 2013 [9]	No	Yes	Yes	Yes	Yes	Moderate
Tosun et al. 2017 [19]	No	Yes	Yes	Yes	Yes	Moderate

## Discussion

Our systematic review and meta-analysis indicate that implants placed simultaneously with autogenous onlay grafts have a survival rate of 93.1% and 86% after a follow-up of <2.5 years and 2.5–5years respectively. Data on implant success is limited ranging from 84.6 to 100% with variable follow-up duration. Analysis of a limited number of studies indicated no significant difference in implant survival between the simultaneous and delayed placement of implants with onlay bone grafts.

Bone augmentation with autogenous onlay grafts has been used for decades in the field of oral implantology. Several systematic reviews have indicated that ridge augmentation using onlay bone grafts is a reliable surgical method for placing implants in ridges where it would otherwise not be possible [5, 33]. A staged treatment procedure consisting of initial bone grafting and implant placement following maturation of the graft is often used in the rehabilitation of deficient alveolar ridges. Simultaneous implant placement with onlay grafts has also been reported, but it has received limited attention in the literature. A 2017 systematic review and meta-analysis by Aghaloo et al. [12] has reported a high implant survival of 85.7 to 100% with delayed placement (8 studies) and a lower implant survival of 73.8 to 91% with simultaneous placement of implants (5 studies) in autogenous onlay bone grafts. However, the study was focussed only on completely edentulous maxillary patients, and it did not conduct a separate meta-analysis for implant survival with simultaneous and delayed implant placement.

Given such deficiency in literature, our meta-analysis presents important results for implantologists practicing onlay bone grafting. On a systematic search of literature with pre-defined inclusion/exclusion criteria, we could identify only 19 studies. The scarcity of literature is an indication that simultaneous implant placement is infrequently practiced with onlay grafts. Our results demonstrated a high pooled survival rate of 93.1% at a follow-up of <2.5years with simultaneous implant placement. However, with a longer follow-up of 2.5–5 years, it dropped to 86%. A high failure rate of >10% after 2.5 years in our meta-analysis is difficult to explain considering our study was a systematic review of prior published literature with different cohorts in different geographical regions. The difference could, however, partly be attributed to the different studies pooled in the two sub-groups based on follow-up duration. In our secondary analysis, we found no difference in implant survival between the simultaneous and delayed placement of implants. This, however, should be interpreted with caution as the 95% CI of the OR was quite wide, and only four studies were available for analysis.

At this stage, it is also important to consider the difference between implant survival and implant success. Implant survival is defined as the proportion of implants still in place at a given follow-up even if they are not in function while implant success takes into account other factors influencing implant function like patient symptoms, peri-implant bone loss, pocket depth, bleeding on probing, and implant mobility [34, 35]. Thus, even if the implant is surviving, it may not necessarily be successful. On descriptive analysis of studies, simultaneous implant placement was associated with a variable success rate of 84.6 to 100% but with a different follow-up duration. Only two comparative studies assessed implant success, and both reported no difference between simultaneous and delayed placement. For deriving strong conclusions, this data needs to be verified by future comparative studies.

Several different sites of autogenous grafts are available providing either membranous or endochondral bone. In our review, iliac and intra-oral grafts were the most commonly used bone grafts. It is known that iliac bone is endochondral in origin while intra-oral grafts are intra-membranous in origin which is similar to the recipient site. Zins et al. [36] have demonstrated that the difference in origin of bone grafts can influence graft resorption rates with faster resorption seen in endochondral grafts. In a large study involving 368 implants, Kang et al. [8] have demonstrated an earlier and higher vertical bone loss with iliac onlay grafts as compared to intra-oral grafts. However, no difference was seen in implant stability and implant survival in their study cohort. In our subgroup analysis based on the type of onlay graft, implant survival was higher with intra-oral grafts (95.7%) as compared to iliac grafts (85.8%). Important to note is that only four studies were available in the sub-group of intra-oral grafts, three of which had a follow-up of 6 months to 1 year.

Other than the type of grafts, several other factors can affect bone resorption with onlay grafts. Vertical augmentations often tend to have higher marginal bone loss as compared to horizontal augmentations [2, 12]. Since there was heterogeneity in the type of defects augmented in the included studies, we further analyzed implant survival based on this variable. Our results demonstrated a higher implant survival with single dimension defects (94.1% with vertical defects and 96.5% with horizontal defects) as compared to combined vertical and horizontal defects (83.3%). Due to the lack of assessment and variability of data presentation, we were unable to analyze the exact changes in marginal bone with simultaneous implant placement.

It has been suggested that simultaneous implant placement with onlay grafts can lead to better osteointegration of implants with limited marginal bone loss. The presence of implants during graft maturation can provide better fixation and stability thereby improving procedural success [25]. Simultaneous implant placement also leads to early loading of the graft. As most of the bone resorption occurs in the first year of grafting, the earlier functional stimulus with simultaneous implant placement may also reduce the crestal bone loss of onlay grafts. This theory was, however, not supported by the results of two comparative studies reporting data on marginal bone loss with both indicating a higher bone loss in the simultaneous implant group.

Our review needs to be interpreted with the following limitations. Foremost, the primary analysis of our study is from single-arm studies with their inherent bias. Only five non-randomized retrospective comparative studies were available with a limited sample size. The overall quality of the included studies was also not high. Secondly, we could only analyze only implant survival and not pool data for implant success and bone loss which are important outcome variables. Thirdly, there was heterogeneity in the included studies owing to differences in follow-up, type of grafts, recipient site, type of implants, etc. A sub-group analysis was attempted to assess the influence of these confounding factors but was restricted by the limited number of available studies. Furthermore, we could not assess the impact of type of implant placed (like butt-joint vs platform switch or smooth vs micro-roughened surfaces) on the outcomes due to lack of details from the included studies. Lastly, majority of the studies did not report the criteria or minimum alveolar dimensions required for simultaneous implant placement. The residual alveolar ridge is a factor of importance for the primary stability of any implant [9]. Difference in implant survival and success between the included studies could have been influenced by this factor.

Nevertheless, our review presents the largest pooled data (1368 implants) of implant survival following simultaneous implant placement with onlay bone grafts. Our study is also the first meta-analysis comparing outcomes of simultaneous and delayed implant placement with onlay grafts. Appropriate sensitivity and sub-group analysis were conducted to present comprehensive evidence to clinicians.

To conclude, data indicate that implants placed simultaneously with autogenous onlay grafts have a survival rate of 93.1% and 86% after a follow-up of <2.5 years and 2.5–5years respectively. Implant success has been assessed sparsely and ranges from 84.6 to 100%. A limited number of studies indicate no significant difference in implant survival between the simultaneous and delayed placement of implants with onlay bone grafts. There is a need for randomized controlled trials comparing simultaneous and delayed implant placement to provide robust evidence. Till then, clinicians should assess each case individually based on the quality of the native bone and its ability to provide primary stability for simultaneous implant placement.

## Supplementary Information

**Additional file 1: Supplementary Table S1.** Search strategy.

## Data Availability

The datasets used and/or analyzed during the current study are available from the corresponding author on reasonable request

## Abbreviations

CI: Confidence intervals
OR: Odds ratio
