# Supplementary material for: Simultaneous implant placement with autogenous onlay bone grafts: a systematic review and meta-analysis
Source: Int J Implant Dent. 2021 Apr 30;7:61. doi: 10.1186/s40729-021-00311-4 (PMC8085156; doi:10.1186/s40729-021-00311-4)
Supplement: Supplementary file 1 — Additional file 1: Supplementary Table S1. Search strategy. [file 40729_2021_311_MOESM1_ESM.docx]

Supplementary Table S1: Search strategy

| Query | Search details |
| --- | --- |
| (dental implant) AND (intraoral graft) | ("dental implants"[MeSH Terms] OR ("dental"[All Fields] AND "implants"[All Fields]) OR "dental implants"[All Fields] OR ("dental"[All Fields] AND "implant"[All Fields]) OR "dental implant"[All Fields]) AND (("intraoral"[All Fields] OR "intraorally"[All Fields]) AND ("graft s"[All Fields] OR "grafted"[All Fields] OR "graftings"[All Fields] OR "transplantation"[MeSH Subheading] OR "transplantation"[All Fields] OR "grafting"[All Fields] OR "transplantation"[MeSH Terms] OR "grafts"[All Fields] OR "transplants"[MeSH Terms] OR "transplants"[All Fields] OR "graft"[All Fields])) |
| (dental implants) AND (calvarial graft) | ("dental implants"[MeSH Terms] OR ("dental"[All Fields] AND "implants"[All Fields]) OR "dental implants"[All Fields]) AND ("calvarial"[All Fields] AND ("graft s"[All Fields] OR "grafted"[All Fields] OR "graftings"[All Fields] OR "transplantation"[MeSH Subheading] OR "transplantation"[All Fields] OR "grafting"[All Fields] OR "transplantation"[MeSH Terms] OR "grafts"[All Fields] OR "transplants"[MeSH Terms] OR "transplants"[All Fields] OR "graft"[All Fields])) |
| (dental implants) AND (block graft) | ("dental implants"[MeSH Terms] OR ("dental"[All Fields] AND "implants"[All Fields]) OR "dental implants"[All Fields]) AND (("block"[All Fields] OR "blocked"[All Fields] OR "blocking"[All Fields] OR "blockings"[All Fields] OR "blocks"[All Fields]) AND ("graft s"[All Fields] OR "grafted"[All Fields] OR "graftings"[All Fields] OR "transplantation"[MeSH Subheading] OR "transplantation"[All Fields] OR "grafting"[All Fields] OR "transplantation"[MeSH Terms] OR "grafts"[All Fields] OR "transplants"[MeSH Terms] OR "transplants"[All Fields] OR "graft"[All Fields])) |
| ((dental implants) AND (ridge augmentation)) AND (simultaneous) | ("dental implants"[MeSH Terms] OR ("dental"[All Fields] AND "implants"[All Fields]) OR "dental implants"[All Fields]) AND (("ridge"[All Fields] OR "ridge s"[All Fields] OR "ridged"[All Fields] OR "ridges"[All Fields] OR "ridging"[All Fields]) AND ("augment"[All Fields] OR "augmentation"[All Fields] OR "augmentations"[All Fields] OR "augmented"[All Fields] OR "augmenting"[All Fields] OR "augments"[All Fields])) AND ("simultaneous"[All Fields] OR "simultaneously"[All Fields]) |
| ((implants) AND (iliac graft)) AND (one-stage) | ("embryo implantation"[MeSH Terms] OR ("embryo"[All Fields] AND "implantation"[All Fields]) OR "embryo implantation"[All Fields] OR "implantation"[All Fields] OR "implant"[All Fields] OR "implant s"[All Fields] OR "implantability"[All Fields] OR "implantable"[All Fields] OR "implantables"[All Fields] OR "implantate"[All Fields] OR "implantated"[All Fields] OR "implantates"[All Fields] OR "implantations"[All Fields] OR "implanted"[All Fields] OR "implanter"[All Fields] OR "implanters"[All Fields] OR "implanting"[All Fields] OR "implantion"[All Fields] OR "implantitis"[All Fields] OR "implants"[All Fields]) AND (("iliacal"[All Fields] OR "iliacs"[All Fields] OR "ilium"[MeSH Terms] OR "ilium"[All Fields] OR "iliac"[All Fields]) AND ("graft s"[All Fields] OR "grafted"[All Fields] OR "graftings"[All Fields] OR "transplantation"[MeSH Subheading] OR "transplantation"[All Fields] OR "grafting"[All Fields] OR "transplantation"[MeSH Terms] OR "grafts"[All Fields] OR "transplants"[MeSH Terms] OR "transplants"[All Fields] OR "graft"[All Fields])) AND "one-stage"[All Fields] |
| ((implants) AND (iliac graft)) AND (immediate) | ("embryo implantation"[MeSH Terms] OR ("embryo"[All Fields] AND "implantation"[All Fields]) OR "embryo implantation"[All Fields] OR "implantation"[All Fields] OR "implant"[All Fields] OR "implant s"[All Fields] OR "implantability"[All Fields] OR "implantable"[All Fields] OR "implantables"[All Fields] OR "implantate"[All Fields] OR "implantated"[All Fields] OR "implantates"[All Fields] OR "implantations"[All Fields] OR "implanted"[All Fields] OR "implanter"[All Fields] OR "implanters"[All Fields] OR "implanting"[All Fields] OR "implantion"[All Fields] OR "implantitis"[All Fields] OR "implants"[All Fields]) AND (("iliacal"[All Fields] OR "iliacs"[All Fields] OR "ilium"[MeSH Terms] OR "ilium"[All Fields] OR "iliac"[All Fields]) AND ("graft s"[All Fields] OR "grafted"[All Fields] OR "graftings"[All Fields] OR "transplantation"[MeSH Subheading] OR "transplantation"[All Fields] OR "grafting"[All Fields] OR "transplantation"[MeSH Terms] OR "grafts"[All Fields] OR "transplants"[MeSH Terms] OR "transplants"[All Fields] OR "graft"[All Fields])) AND ("immediate"[All Fields] OR "immediately"[All Fields]) |
| ((implants) AND (iliac graft)) AND (simultaneous) | ("embryo implantation"[MeSH Terms] OR ("embryo"[All Fields] AND "implantation"[All Fields]) OR "embryo implantation"[All Fields] OR "implantation"[All Fields] OR "implant"[All Fields] OR "implant s"[All Fields] OR "implantability"[All Fields] OR "implantable"[All Fields] OR "implantables"[All Fields] OR "implantate"[All Fields] OR "implantated"[All Fields] OR "implantates"[All Fields] OR "implantations"[All Fields] OR "implanted"[All Fields] OR "implanter"[All Fields] OR "implanters"[All Fields] OR "implanting"[All Fields] OR "implantion"[All Fields] OR "implantitis"[All Fields] OR "implants"[All Fields]) AND (("iliacal"[All Fields] OR "iliacs"[All Fields] OR "ilium"[MeSH Terms] OR "ilium"[All Fields] OR "iliac"[All Fields]) AND ("graft s"[All Fields] OR "grafted"[All Fields] OR "graftings"[All Fields] OR "transplantation"[MeSH Subheading] OR "transplantation"[All Fields] OR "grafting"[All Fields] OR "transplantation"[MeSH Terms] OR "grafts"[All Fields] OR "transplants"[MeSH Terms] OR "transplants"[All Fields] OR "graft"[All Fields])) AND ("simultaneous"[All Fields] OR "simultaneously"[All Fields]) |
| (implants) AND (onlay grafts) | ("embryo implantation"[MeSH Terms] OR ("embryo"[All Fields] AND "implantation"[All Fields]) OR "embryo implantation"[All Fields] OR "implantation"[All Fields] OR "implant"[All Fields] OR "implant s"[All Fields] OR "implantability"[All Fields] OR "implantable"[All Fields] OR "implantables"[All Fields] OR "implantate"[All Fields] OR "implantated"[All Fields] OR "implantates"[All Fields] OR "implantations"[All Fields] OR "implanted"[All Fields] OR "implanter"[All Fields] OR "implanters"[All Fields] OR "implanting"[All Fields] OR "implantion"[All Fields] OR "implantitis"[All Fields] OR "implants"[All Fields]) AND (("inlays"[MeSH Terms] OR "inlays"[All Fields] OR "onlay"[All Fields] OR "onlays"[All Fields] OR "onlayed"[All Fields]) AND ("graft s"[All Fields] OR "grafted"[All Fields] OR "graftings"[All Fields] OR "transplantation"[MeSH Subheading] OR "transplantation"[All Fields] OR "grafting"[All Fields] OR "transplantation"[MeSH Terms] OR "grafts"[All Fields] OR "transplants"[MeSH Terms] OR "transplants"[All Fields] OR "graft"[All Fields])) |
